# Evaluation of Hospital Cesarean Delivery–Related Profits and Rates in the United States

**DOI:** 10.1001/jamanetworkopen.2021.2235

**Published:** 2021-03-19

**Authors:** Rie Sakai-Bizmark, Michael G. Ross, Dennys Estevez, Lauren E. M. Bedel, Emily H. Marr, Yusuke Tsugawa

**Affiliations:** 1 Lundquist Institute for Biomedical Innovation at Harbor-UCLA Medical Center, Torrance, California; 2 Department of Pediatrics, Harbor-UCLA Medical Center and David Geffen School of Medicine, University of California, Los Angeles, Torrance; 3 Department of Obstetrics and Gynecology, Harbor-UCLA Medical Center and David Geffen School of Medicine, University of California, Los Angeles, Torrance; 4 Division of General Internal Medicine and Health Services Research, David Geffen School of Medicine, University of California, Los Angeles; 5 Department of Health Policy and Management, Fielding School of Public Health, University of California, Los Angeles

## Abstract

**Importance:**

A high cesarean delivery rate in US hospitals indicates the potential overuse of this procedure; however, underlying causes of the excessive use of cesarean procedures in the US have not been fully understood.

**Objective:**

To investigate the association between the probability of cesarean delivery at the patient-level and profit per procedure from cesarean deliveries.

**Design, Setting, and Participants:**

This observational, cross-sectional study used a nationally representative sample of hospital discharge data from women at low risk for cesarean birth who delivered newborns between 2010 and 2014 in the US. Data were gathered from the Nationwide Readmissions Database from the Healthcare Cost and Utilization Project, compiled by the Agency for Healthcare Research and Quality. Data cleaning and analyses were conducted between August 2019 and May 2020.

**Exposures:**

Hospital-level median value of profits from cesarean deliveries, defined as the difference between the charge and the cost for cesarean delivery calculated for each hospital.

**Main Outcomes and Measures:**

Our primary outcome was the individual-level probability of undergoing a cesarean delivery. We examined the association with the hospital-level median value of profits per procedure for cesarean delivery (defined as the difference between the charge and the cost for cesarean delivery) using hierarchical regression models adjusted for patient and hospital characteristics and year-fixed effects.

**Results:**

A total of 13 215 853 deliveries were included in our analyses (mean [SE] age, 27.4 [0] years), of which 2 202 632 (16.7%) were cesarean deliveries. After adjusting for potential confounders, pregnant women were more likely to have a cesarean birth when they delivered at hospitals with higher profits per procedure from cesarean deliveries. Women cared for at hospitals with the highest (adjusted odds ratio, 1.08; 95% CI, 1.02-1.14; *P* = .005) and second-highest profit quartiles (adjusted odds ratio, 1.07; 95% CI, 1.02-1.13; *P* = .007) had higher probabilities of a cesarean delivery compared with those cared for at hospitals in the lowest profit quartile.

**Conclusions and Relevance:**

In this cross-sectional study of US nationally representative hospital discharge data, hospitals with higher profits per cesarean procedure were associated with an increased probability of delivering newborns through cesarean birth. These findings highlight the potential influence financial incentives play in determining a high cesarean delivery rate in the US.

## Key Points

**Question:**

Do patients who receive care at hospitals that make higher profits have a higher probability of cesarean delivery compared with hospitals that make less profits?

**Findings:**

In this cross-sectional, nationally representative study using hospital discharge data between 2010 and 2014, women delivering at hospitals with higher profits per procedure were associated with an increased probability of undergoing cesarean delivery.

**Meaning:**

These findings suggest an association between financial incentives and the US cesarean delivery rate.

## Teaser

This cross-sectional study of discharge data from a national database of US hospitals examines the association of profits hospitals received from cesarean procedures and rates of cesarean delivery.

## Introduction

Cesarean delivery is the most frequent surgical procedure performed in the US.^1,2,3^ Nationally, the procedure accounts for 1 in 3 births.^1,4,5,6^ Cesarean delivery is a potentially lifesaving intervention that is necessary in some cases to protect the lives and health of the mother and newborn. Compared with vaginal delivery, however, the procedure is associated with a higher risk of various maternal complications, such as maternal infection and subsequent pregnancy complications,^7,8,9,10,11,12,13,14,15,16^ and a higher likelihood of rehospitalization within 6 weeks of delivery.^17^ Furthermore, the likelihood of maternal morbidity increases for mothers who repeatedly undergo cesarean delivery.^18,19,20^

Healthy People 2020—a framework designed to address health disparities across the US—calls for a 10% relative decrease in primary cesarean deliveries among low-risk women, with the goal of lowering the cesarean rate to 23.9%.^21^ While an official midprogram review is not yet available, it is estimated that the US will not meet this goal.^1,3,4,5,6,22^ According to the World Health Organization, cesarean delivery rates above 10% to 15% are generally not associated with improvements in maternal, neonatal, and infant mortality rates,^23^ though a previous study suggested a cesarean rate of 19% to be more attainable.^24^ A high rate of cesarean deliveries in the US indicates a potential overuse of this procedure; however, underlying causes of the excessive use of cesarean delivery in the US have not been fully understood.

Many studies have investigated the impact of financial incentives on individual obstetricians, suggesting that financial incentives may be influential in increasing the likelihood of cesarean delivery.^2,25,26^ A 2017^25^ study found that an increase in physician or hospital price by a single standard deviation resulted in 12% and 31% increases in the likelihood of a cesarean delivery, respectively. Another study reported cesarean rates across US communities varied by more than 16-fold.^2^ Although it is possible that hospitals and physicians may respond differently to financial incentives, and optimal design of policies differ based on how financial incentives affect the 2 groups, evidence is limited as to how financial incentives for hospitals are associated with the rate of cesarean delivery. Furthermore, existing research on this topic is outdated and no studies exist that have investigated how hospital incentives factor into the rate of cesarean deliveries using contemporary, national data.^27,28^

To address this important knowledge gap, we investigated the association between the probability of undergoing a cesarean delivery at the patient-level and profit per procedure from cesarean deliveries using national data on hospital discharges in the US. In this study, profits were defined as the difference between charged prices and costs.

## Methods

We used the Healthcare Cost and Utilization Project’s (HCUP) Nationwide Readmissions Database (NRD) for years 2010 through 2014, compiled by the Agency for Healthcare Research and Quality (AHRQ).^29^ The NRD has been used previously to explore associations between maternal health and delivery outcomes as they relate to hospital characteristics.^30,31^ Data analyses were performed between August 2019 and May 2020. This study followed the Strengthening and Reporting of Observational Studies in Epidemiology (STROBE) reporting guideline for cross-sectional studies. The Lundquist Institute for Biomedical Innovation at Harbor-UCLA Medical Center institutional review board approved this study as exempt because it used deidentified data.

### Identification of Patients

We identified cesarean and vaginal deliveries based on *International Classification of Diseases, Ninth Revision, Clinical Modification (ICD-9-CM)*.^32,33,34^ We limited our analytic cohort to women at low risk for a cesarean delivery.^35^ We excluded hospitals that did not perform cesarean deliveries in order to exclude birthing facilities that do not have the capability of providing cesarean deliveries^30^ and hospitals that performed less than 100 deliveries per year to avoid unstable estimates, an approach used in prior studies.^1,2,30,34,35^

### Exposure Variable

Our primary exposure variable was the hospital-level median value of cesarean procedure profits. Once cesarean deliveries were identified, we used the difference between charges and costs to approximate the profit per procedure each hospital makes by providing a cesarean delivery.^36^ We defined charges as the amount hospitals billed for services; this differs from costs, which are the actual expenses incurred throughout the production of hospital services, such as wages, supplies, and utility. Total charges tracked by HCUP exclude professional (ie, physician) fees.^37^ To estimate costs, we multiplied charges by the hospital-level cost-to-charge ratio (CCR).^38^ Each file contains hospital-specific CCRs based on all-payer inpatient costs for nearly every hospital in the NRD.^37^ CCRs are calculated by dividing inpatient costs by inpatient charges for each individual hospital. Cost information was obtained from hospital accounting reports collected by the Centers for Medicare & Medicaid Services. Therefore, each hospital has a single CCR, which is not specific to each procedure.^37^ CCRs allow researchers to approximate costs across facilities, as each hospital’s costs vary.

We estimated the expected charge and cost (Ŷ) adjusted by individual characteristics (see Outcome and Adjustment Variables in Methods) using a Poisson regression model.^39^ To account for inflation, we used the medical care component of the Consumer Price Index to convert charges and costs to 2015 US dollars.^40^ We calculated the difference between charge and cost for cesarean deliveries for each patient, and estimated the median value for each hospital. We then categorized hospitals into 4 equal-sized groups (quartiles) based on estimated profits.

### Outcome and Adjustment Variables

The outcome variable was the probability of cesarean delivery, measured at the patient level. Patient-level variables included age (<18 years, 18-35 years, 36-40 years, 41-45 years, and >45 years), insurance type (public, private, self-pay, or other), median income quartile (based on the patient’s residential zip code), maternal comorbidities, delivery-related procedures, and delivery and postpartum complications that affect the likelihood of women undergoing a cesarean delivery (used in previous studies^17,33,41,42,43^) (Table 1). Hospital-level variables included hospital location (urban vs rural), teaching status, bed size (small, medium, or large), and hospital ownership (government, nonprofit private, or investor-owned private). We used year fixed effects to account for secular trend of the cesarean delivery rate.

**Table 1.** Maternal Morbidity

Maternal comorbidities
Asthma
Diabetes
Drug dependency/substance abuse
Hypertensive disorder
Obesity
Psychiatric disease
Pregnancy-related hypertensive disorder
Seizure disorder
Smoking
Thyroid disease
**Delivery-related procedures**
Forceps delivery
Vacuum delivery
Operative delivery (unspecified)
Induction of labor
Episiotomy
Hysterectomy
Transfusion
Dilation and curettage
Laparotomy
**Delivery complications**
Hemorrhage
Infection
Laceration
Operative injury
Thrombotic event
Uterine rupture
Other delivery complications
**Postpartum complications**
Acute cardiovascular disease (eg, cardiomyopathy, heart failure, myocardial infarction)
Acute cerebrovascular disease (eg, stroke and intracranial hemorrhage)
Anesthesia complications
Appendicitis
Bacteremia, sepsis
Gallbladder disease
Nonspecific postpartum diagnosis
Hemorrhage and/or retained products of conception
Hypertensive disorder
Mastitis, breast abscess
Pancreatitis
Psychiatric disease (eg, substance abuse)
Thrombotic event
Upper respiratory infection
Urinary tract infection (including pyelonephritis)
Uterine infection
Wound infection and/or breakdown

### Statistical Analyses

We first described the demographic and clinical characteristics of the study population, as well as the hospital characteristics by profit quartiles for cesarean delivery at the individual patient-level. We then examined the association between hospitals’ profit per procedure for cesarean deliveries and the probability of cesarean deliveries. Given that the outcome was a binary variable (whether women delivered via cesarean or vaginal birth) and there was potential correlation between women cared for at the same hospital, we used hierarchical logistic regression, adjusted for the patient- and hospital-level variables and year fixed effects. We used the survey analysis command sets to account for sample weights in variance calculations as recommended by HCUP.

We then estimated changes in the expected number of cesarean deliveries if all mothers in the US had delivered at hospitals in the lowest profit quartile for cesarean births. In doing so, we first calculated the probability of undergoing a cesarean delivery for every individual with a delivery record (Ŷ). We obtained the expected number of cesarean deliveries by summing these probabilities. Then, we created a hypothetical data set, one in which all women delivered at the lowest profit quartile category, and ran the same procedure to obtain the expected number of cesarean deliveries under this scenario. We defined the difference between these 2 expected numbers as avoidable cesarean deliveries.

### Sensitivity Analyses

We conducted a series of sensitivity analyses. Profits were considered a continuous variable. As profits were highly skewed, the log-transformed version of the continuous profit variable was included in the model. The difference in cost between cesarean and vaginal deliveries was used to represent the profit for cesarean procedures. We then reanalyzed the data, restricted to hospitals with 50 or more deliveries per year, rather than 100 or more deliveries per year as in our main analyses, to test whether our findings were sensitive to our choice of the minimum threshold of hospital volume. Fourth, because hospital identifiers do not track the same hospital across calendar years, we used only 2014 data in the analyses. We considered a 2-sided *P* value <0.05 statistically significant. We conducted all analyses using SAS version 9.4 (SAS Institute Inc).

## Results

Our final analytic sample consisted of 13 215 853 deliveries (mean [SE] age, 27.4 [0] years), of which 2 202 632 (16.7%) were cesarean deliveries. The cesarean rate across profit quartiles varied from 15.6% (368 718 of 2 356 544 deliveries) to 17.6% (593 365 of 3 368 493 deliveries) in the lowest and highest quartiles, respectively (Table 2). Cesarean delivery profits varied from $4969 for the lowest profit quartile compared with $26 129 for the highest profit quartile. Frequencies and percentages of individuals with maternal comorbidities, delivery-related procedures, delivery complications, and postpartum complications across all 4 profit quartiles are shown in eTable in the Supplement. We found that individual patients cared for at hospitals with high profits per procedure from cesarean deliveries were older (mean [SE] age: 27.8 [0.1] years vs lowest profit quartile, 27.1 [0.1] years), more likely to be covered by public health insurance (1 545 055 deliveries [45.9%] vs 987 009 deliveries [41.9%]), less likely to have delivery complications (465 247 deliveries [13.8%] vs 355 827 deliveries [15.1%]), and more likely to have postpartum complications (80 677 deliveries [2.4%] vs 51 405 deliveries [2.2%]) compared with patients cared for at low-profit hospitals (Table 2).

**Table 2.** Patient Characteristics by Hospital Profit Quartile

Characteristics	Deliveries, No. (%)					*P* value
	Total (N = 13 215 853)	Profit quartiles				
		Lowest (n = 2 356 544)	Quartile 2 (n = 3 400 263)	Quartile 3 (n = 4 090 553)	Highest (n = 3 368 493)	
Total cesarean deliveries	2 202 632 (16.7)	368 718 (15.6)	536 480 (15.8)	704 070 (17.2)	593 365 (17.6)	<.001
Age, mean (SE), y	27.4 (0)	27.1 (0.1)	27.0 (0.1)	27.5 (0.1)	27.8 (0.1)	<.001
Insurance						
Public	5 754 610 (43.5)	987 009 (41.9)	1 448 528 (42.6)	1 774 018 (43.4)	1 545 055 (45.9)	<.001
Private	6 776 950 (51.3)	1 213 442 (51.5)	1 751 315 (51.5)	2 122 918 (51.9)	1 689 275 (50.1)	
Self-pay	209 999 (1.6)	43 588 (1.8)	61 653 (1.8)	63 255 (1.5)	41 504 (1.2)	
Other	442 793 (3.4)	98 860 (4.2)	131 822 (3.9)	122 850 (3.0)	89 261 (2.6)	
Morbidity^a^						
Maternal comorbidities	3 361 170 (25.4)	5,77 594 (24.5)	847 279 (24.9)	1 091 359 (26.7)	844 939 (25.1)	.03
Delivery-related procedures	6 395 614 (48.4)	1 127 803 (47.9)	1 694 831 (49.8)	1 997 077 (48.8)	1 575 903 (46.8)	.004
Delivery complications	1 959 591 (14.8)	355 827 (15.1)	517 844 (15.2)	620 673 (15.2)	465 247 (13.8)	.002
Postpartum complications	305 099 (2.3)	51 405 (2.2)	76 247 (2.2)	96 769 (2.4)	80 677 (2.4)	.002

^a^ Frequencies and percentages for specific causes associated with this category are shown in the eTable in the Supplement.

### Hospital Profit and Cesarean Delivery Rate

After adjusting for potential confounders, we found that delivering at the highest (adjusted odds ratio [aOR], 1.08; 95% CI, 1.02-1.14; *P* = .005) and the second-highest profit quartiles (aOR, 1.07; 95% CI, 1.02-1.13; *P* = .007) was associated with increased odds of a cesarean delivery compared with delivering at hospitals in the lowest profit quartile (Table 3). All the variables employed in the models were statistically significantly associated with the probability of undergoing a cesarean birth.

**Table 3.** Adjusted Association Between Hospital Profit for Cesarean Deliveries and Patient Cesarean Delivery Rate^a^

Hospital profits quartile	Adjusted odds ratio (95% CI)	*P* value
1st (lowest)	1 [Reference]	NA
2nd	0.99 (0.93-1.04)	.63
3rd	1.07 (1.02-1.13)	.007
4th (highest)	1.08 (1.02-1.14)	.005

Abbreviation: NA, not applicable.

^a^ Adjusted for patient characteristics (patients’ age, insurance type, median income quartile based on residential zip code, maternal comorbidities, delivery-related procedures, and delivery and postpartum complications), hospital characteristics (location, teaching status, bed size, and ownership), and year fixed effects.

We estimated that if all women were to be cared for at hospitals in the lowest profit quartile, 146 752 fewer cesarean deliveries (a 6.7% reduction) would have been performed throughout the entire study period. All sensitivity analyses mirrored our main analyses. (Table 4)

**Table 4.** Adjusted Association Between Hospital Profit for Cesarean Deliveries and Patient Cesarean Delivery Rate^a^

Hospital profit quartile	Adjusted odds ratio (95% CI)	*P* value
Log of gain (continuous)^b^	1.03 (1.01-1.06)	.01
Difference in cost between cesarean and vaginal delivery^c^		
1st (lowest)	1 [Reference]	NA
2nd	1.95 (1.81-2.11)	<.001
3rd	1.83 (1.69-1.99)	<.001
4th (highest)	1.58 (1.46-1.72)	<.001
Excluding hospitals <50 deliveries^d^		
1st (lowest)	1 [Reference]	NA
2nd	0.98 (0.93-1.04)	.54
3rd	1.09 (1.03-1.14)	.002
4th (highest)	1.09 (1.03-1.15)	.002
2014 only^e^		
1st (lowest)	1 [Reference]	NA
2nd	0.95 (0.85-1.05)	.31
3rd	0.98 (0.89-1.09)	.72
4th (highest)	1.09 (0.98-1.21)	.12

^a^ Adjusted for patient characteristics (patients’ age, insurance type, median income quartile based on residential zip code, maternal comorbidities, delivery-related procedures, and delivery and postpartum complications), hospital characteristics (location, teaching status, bed size, and ownership), and year fixed effects.

^b^ Profits were used as a continuous variable. Because profits were highly skewed, the log-transformed version of the continuous profit variable was included in the model.

^c^ The difference in cost between cesarean and vaginal deliveries was used to represent the profit for cesarean procedures.

^d^ Hospitals with 50 or more deliveries per year were used, rather than 100 or more deliveries per year, to test whether findings were sensitive to the minimum threshold of hospital volume used in the study.

^e^ Only 2014 data were used in this analysis.

## Discussion

Using nationally representative data of pregnant women who delivered in the US, we found an association between hospital cesarean delivery profits and the likelihood of women delivering via cesarean. We found a shockingly sizeable, 5.3-fold difference in hospital cesarean delivery profits between the lowest profit quartile and the highest profit quartile. A previous study^44^ found a 2.2-fold difference in costs for deliveries (vaginal and cesarean), although this study did not examine profits and the difference in costs varied more widely for cesarean deliveries and was likely influenced by length of stay. The authors also found that costs were higher at facilities with higher rates of cesarean delivery.^44^ Our finding illuminates the important association financial incentives have on hospitals, as a potential “upstream” factor for the high number of cesarean deliveries performed in the US. Our findings should be informative for policymakers and payers to design interventions that can effectively reduce unnecessary cesarean deliveries.

Although many studies have investigated the impact of financial incentives on physicians’ decisions to deliver via cesarean, evidence is limited as to whether higher hospital profits from cesarean births affect hospitals’ delivery of care. Existing studies on procedures in other fields of medicine, such as newer prostate cancer treatment technology and multiple cardiovascular procedures, indicate that higher payments, among other sometimes unidentifiable factors, may be associated with a higher number of procedures provided.^45,46,47^ To our knowledge, our study is one of the first to show the association between financial incentives and the number of cesarean deliveries provided at individual hospitals.

As high rates of cesarean deliveries have increasingly attracted attention from policymakers, several states implemented bundled payments for perinatal care under Medicaid programs with the intent of reducing financial incentives that could encourage the use of cesarean deliveries.^48^ However, evidence is mixed as to whether such payment reforms effectively change the provision of cesarean deliveries.^49,50^

The mechanisms through which hospitals’ profits may factor into the decision-making process of front-line physicians are complex. Although the ultimate decision to perform a cesarean birth is made between patient and physician, there are multiple extraneous factors that may be associated with either party’s decision.^2^ For health systems where physicians’ incomes are tied to procedure quantity (eg, physicians bill directly to payers for physicians fees, or an individual physician’s salary is tied to revenues generated by those fees through bonuses), physician behaviors are likely associated with such financial incentives. More cesarean procedures would produce a bigger paycheck. More importantly, even within health systems where physicians’ incomes are not directly tied to the profit of health systems, higher profit hospitals may potentially institute a culture and structure (via medical staff, nursing) associated with childbirth delivery, or choose to implement programs (eg, quality assessment, statistical reporting) that are correlated with the reduction of high cesarean delivery rates.^51^ It is also possible that hospital profits may be a marker of other factors correlated with its cesarean delivery rate, such as the preferences of mothers living in an area where a given hospital is located. A better understanding of the dynamics and medical culture that may be associated with hospital cesarean delivery profits and the US cesarean birth rate is instrumental in the design of future policies that can effectively reduce the rate of unnecessary cesarean deliveries.

### Limitations

This study had several limitations. First, as is the case with any observational study, we could not eliminate the possibility of the existence of unmeasured confounders. Given that we used administrative claims data, detailed clinical information beyond *ICD-9-CM* codes on mothers’ medical records was unavailable. Though we adjusted our models to account for maternal and hospital demographic characteristics, certain maternal comorbidities, delivery-related procedures, and delivery and postpartum complications, mothers (and fetuses) who are cared for at hospitals with higher cesarean delivery profits may experience additional complications unmeasured by our particular data set compared with those cared for at hospitals with lower profits from cesarean procedures, and therefore experienced a higher likelihood of cesarean delivery. For example, had we been able to identify nulliparous women, we would have been able to further refine our selection criteria for identifying women at low risk of cesarean deliveries. Similarly, as the NRD does not include state or regional information, we were unable to examine the association between geographic location and hospital profits. It is possible that the impact of financial incentives on practice patterns differ between hospitals and individual physicians. Our analysis focused on hospital behavior, and we did not assess direct possible competing interests for physicians, between the decision to perform a cesarean and their personal interests (ie, fees).

Second, it was possible that hospitals with a large number of cesarean deliveries had stronger bargaining power to negotiate with insurers, and ultimately received higher payments per procedure^52^ (ie, reverse causality), rather than profits from the cesarean procedure increasing the probability of cesarean delivery. Third, charges may not represent the payment that hospitals actually receive^27,53,54^; therefore, the difference between charge and cost may not represent net profits. However, prior research has shown that hospital charges correlate well with revenue,^55^ which supports the use of the difference between charge and cost as a proxy for profit in our study. Additionally, we included another indicator, the difference between cost for cesarean and vaginal deliveries, to represent the profit hospitals gain from performing cesarean procedures. Inclusion of this indicator showed similar results. Fourth, although we detected a statistically significant difference across hospitals with different profit levels from cesarean procedures, the clinical significance is still questionable, especially because of the small differences between hospitals with different levels of profits. In addition, the dose-response relationship between hospital profit for cesarean procedures and cesarean delivery rates observed in our main analysis was not observed in our sensitivity analysis. Further research is warranted to understand the clinical magnitude of hospital profits, and what interventions are effective in reducing unnecessary cesarean deliveries in the US.

Fifth, as individual patient and hospital identifiers are not coded consistently across calendar years, individuals who had multiple deliveries throughout the study period could have been included in the analyses more than once. In the same way, multiple individuals from the same hospitals could have been included in the analyses. To account for the issue of potential correlation owing to repeated hospital observation, we used multi-level models in the analyses (ie, the survey commands accounted for the multi-level nature of the data) and included year fixed effect (ie, indicator variables for the year of the data) in our regression models allowing us to effectively compare hospitals within the same year. Additionally, we categorized hospitals into groups based on their profit level for each year (it was possible that the same hospital could be categorized as a high-profit hospital in one year and a low-profit hospital in the following year). Therefore, residual correlations because of repeated hospital observation had limited impact. However, these methods may only partially account for the potential correlation of women treated at the same hospital. To test whether our findings were sensitive to this issue, we conducted a sensitivity analysis restricting our sample to observations from a single year.

Sixth, we estimated cost data by multiplying charges by the hospital-level CCR. We were unable to determine to what extent cesarean deliveries contribute to the estimation of CCR; however, cesarean delivery is the most frequent surgical procedure performed in the US.^1,2,3^ Therefore, cesarean deliveries may be an important contributor in the estimation of hospitals’ CCRs.

## Conclusions

This cross-sectional study of US national hospital discharge data found that delivering at hospitals with higher profits from cesarean procedures was associated with a higher likelihood of patients undergoing a cesarean delivery compared with patients who delivered at lower-profit hospitals. These findings suggest that financial incentives could be associated with variations in the rate of cesarean deliveries across the US. A greater understanding of the dynamics that contribute to the relationship between hospital profit and cesarean delivery rates may assist in future steps taken to reduce the rate of unnecessary cesarean procedures.

## Notes

Supplement. **eTable.** Frequencies and Percentages Associated With Maternal Comorbidities, Delivery-Related Procedures, Delivery Complications, and Postpartum Complications

## References

[1] Kozhimannil KB, Law MR, Virnig BA. Cesarean delivery rates vary tenfold among US hospitals; reducing variation may address quality and cost issues. Health Aff (Millwood). 2013;32(3):527-535. doi:10.1377/hlthaff.2012.1030 23459732 PMC3615450

[2] Little SE, Orav EJ, Robinson JN, Caughey AB, Jha AK. The relationship between variations in cesarean delivery and regional health care use in the United States. Am J Obstet Gynecol. 2016;214(6):735.e1-735.e8. doi:10.1016/j.ajog.2015.12.023 26721777

[3] Martin JA, Hamilton BE, Osterman MJK, Driscoll AK. Births: Final Data for 2018. Natl Vital Stat Rep. 2019;68(13):1-47. 32501202

[4] Osterman MJ, Martin JA. Trends in low-risk cesarean delivery in the United States, 1990-2013. Natl Vital Stat Rep. 2014;63(6):1-16. 25383560

[5] Menacker F, Hamilton BE. Recent trends in cesarean delivery in the United States. NCHS Data Brief. 2010;(35):1-8. 20334736

[6] Martin JA, Hamilton BE, Osterman MJK, Driscoll AK, Drake P. Births: Final Data for 2017. Natl Vital Stat Rep. 2018;67(8):1-50. 30707672

[7] Blondon M, Casini A, Hoppe KK, Boehlen F, Righini M, Smith NL. Risks of venous thromboembolism after cesarean sections: a meta-analysis. Chest. 2016;150(3):572-596. doi:10.1016/j.chest.2016.05.021 27262227

[8] de la Cruz CZ, Thompson EL, O’Rourke K, Nembhard WN. Cesarean section and the risk of emergency peripartum hysterectomy in high-income countries: a systematic review. Arch Gynecol Obstet. 2015;292(6):1201-1215. doi:10.1007/s00404-015-3790-2 26104125

[9] Deneux-Tharaux C, Carmona E, Bouvier-Colle MH, Bréart G. Postpartum maternal mortality and cesarean delivery. Obstet Gynecol. 2006;108(3 Pt 1):541-548. doi:10.1097/01.AOG.0000233154.62729.24 16946213

[10] Kainu JP, Halmesmäki E, Korttila KT, Sarvela PJ. Persistent pain after cesarean delivery and vaginal delivery: a prospective cohort study. Anesth Analg. 2016;123(6):1535-1545. doi:10.1213/ANE.0000000000001619 27870738

[11] Keag OE, Norman JE, Stock SJ. Long-term risks and benefits associated with cesarean delivery for mother, baby, and subsequent pregnancies: systematic review and meta-analysis. PLoS Med. 2018;15(1):e1002494. doi:10.1371/journal.pmed.1002494 29360829 PMC5779640

[12] Leth RA, Møller JK, Thomsen RW, Uldbjerg N, Nørgaard M. Risk of selected postpartum infections after cesarean section compared with vaginal birth: a five-year cohort study of 32 468 women. Acta Obstet Gynecol Scand. 2009;88(9):976-983. doi:10.1080/00016340903147405 19642043

[13] Lydon-Rochelle M, Holt VL, Martin DP, Easterling TR. Association between method of delivery and maternal rehospitalization. JAMA. 2000;283(18):2411-2416. doi:10.1001/jama.283.18.2411 10815084

[14] Rossen J, Okland I, Nilsen OB, Eggebø TM. Is there an increase of postpartum hemorrhage, and is severe hemorrhage associated with more frequent use of obstetric interventions? Acta Obstet Gynecol Scand. 2010;89(10):1248-1255. doi:10.3109/00016349.2010.514324 20809871

[15] Tarney CM. Bladder injury during cesarean delivery. Curr Womens Health Rev. 2013;9(2):70-76. doi:10.2174/157340480902140102151729 24876830 PMC4033551

[16] Temming LA, Raghuraman N, Carter EB, . Impact of evidence-based interventions on wound complications after cesarean delivery. Am J Obstet Gynecol. 2017;217(4):449.e1-449.e9. doi:10.1016/j.ajog.2017.05.070 28601567 PMC5614824

[17] Belfort MA, Clark SL, Saade GR, . Hospital readmission after delivery: evidence for an increased incidence of nonurogenital infection in the immediate postpartum period. Am J Obstet Gynecol. 2010;202(1):35.e1-35.e7. doi:10.1016/j.ajog.2009.08.029 19889389

[18] Marshall NE, Fu R, Guise JM. Impact of multiple cesarean deliveries on maternal morbidity: a systematic review. Am J Obstet Gynecol. 2011;205(3):262.e1-262.e8. doi:10.1016/j.ajog.2011.06.035 22071057

[19] Silver RM, Landon MB, Rouse DJ, ; National Institute of Child Health and Human Development Maternal-Fetal Medicine Units Network. Maternal morbidity associated with multiple repeat cesarean deliveries. Obstet Gynecol. 2006;107(6):1226-1232. doi:10.1097/01.AOG.0000219750.79480.84 16738145

[20] Sondgeroth KE, Wan L, Rampersad RM, . Risk of maternal morbidity with increasing number of cesareans. Am J Perinatol. 2019;36(4):346-351. doi:10.1055/s-0038-1673653 30372778

[21] US Department of Health and Human Services. Maternal, Infant, and Child Health, 7.1: reduce cesarean births among low-risk women with no prior cesarean births. Updated 2019. Accessed October 24, 2019. https://www.healthypeople.gov/node/4900/data_details

[22] Maternal Safety Foundation. NTSV Rate Dashboard. 2018. Accessed October 24, 2019. https://www.cesareanrates.org/ntsvdashboard

[23] Betran AP, Torloni MR, Zhang JJ, Gülmezoglu AM; World Health Organization Working Group on Caesarean Section. WHO statement on caesarean section rates. BJOG. 2016;123(5):667-670. doi:10.1111/1471-0528.13526 26681211 PMC5034743

[24] Molina G, Weiser TG, Lipsitz SR, . Relationship between cesarean delivery rate and maternal and neonatal mortality. JAMA. 2015;314(21):2263-2270. doi:10.1001/jama.2015.15553 26624825

[25] Foo PK, Lee RS, Fong K. Physician prices, hospital prices, and treatment choice in labor and delivery. Am J Health Econ. 2017;3(3):422-453. doi:10.1162/ajhe_a_00083

[26] Alexander D. Does physician pay affect procedure choice and patient health? evidence from Medicaid c-section use. Working Paper 2017-07. Federal Reserve Bank of Chicago; 2017.

[27] Keeler EB, Brodie M. Economic incentives in the choice between vaginal delivery and cesarean section. Milbank Q. 1993;71(3):365-404. doi:10.2307/3350407 8413067

[28] Grytten J, Monkerud L, Hagen TP, Sørensen R, Eskild A, Skau I. The impact of hospital revenue on the increase in caesarean sections in Norway: a panel data analysis of hospitals 1976-2005. BMC Health Serv Res. 2011;11:267. doi:10.1186/1472-6963-11-267 21992174 PMC3210106

[29] Healthcare Cost and Utilization Project. HCUP Nationwide Readmissions Database, 2010-2014. Accessed August 5, 2019. https://www.hcup-us.ahrq.gov/nrdoverview.jsp

[30] Clapp MA, James KE, Melamed A, Ecker JL, Kaimal AJ. Hospital volume and cesarean delivery among low-risk women in a nationwide sample. J Perinatol. 2018;38(2):127-131. doi:10.1038/jp.2017.173 29120454

[31] Federspiel JJ, Suresh SC, Darwin KC, Szymanski LM. Hospitalization duration following uncomplicated cesarean delivery: predictors, facility variation, and outcomes. AJP Rep. 2020;10(2):e187-e197. doi:10.1055/s-0040-1709681 32577322 PMC7305021

[32] Kuklina EV, Whiteman MK, Hillis SD, . An enhanced method for identifying obstetric deliveries: implications for estimating maternal morbidity. Matern Child Health J. 2008;12(4):469-477. doi:10.1007/s10995-007-0256-6 17690963

[33] Asch DA, Nicholson S, Srinivas S, Herrin J, Epstein AJ. Evaluating obstetrical residency programs using patient outcomes. JAMA. 2009;302(12):1277-1283. doi:10.1001/jama.2009.1356 19773562

[34] Easter SR, Robinson JN, Carusi D, Little SE. The US twin delivery volume and association with cesarean delivery rates: a hospital-level analysis. Am J Perinatol. 2018;35(4):345-353. doi:10.1055/s-0037-1607316 29020694

[35] Armstrong JC, Kozhimannil KB, McDermott P, Saade GR, Srinivas SK; Society for Maternal-Fetal Medicine Health Policy Committee. Comparing variation in hospital rates of cesarean delivery among low-risk women using 3 different measures. Am J Obstet Gynecol. 2016;214(2):153-163. doi:10.1016/j.ajog.2015.10.935 26593970

[36] Anderson GF. From ‘soak the rich’ to ‘soak the poor’: recent trends in hospital pricing. Health Aff (Millwood). 2007;26(3):780-789. doi:10.1377/hlthaff.26.3.780 17485757

[37] Healthcare Cost and Utilization Project. Cost-to-charge ratio for inpatient files, 2020. Accessed August 6, 2020. https://www.hcup-us.ahrq.gov/db/ccr/ip-ccr/ip-ccr.jsp

[38] Healthcare Cost and Utilization Project. Cost-to-charge ratio files, 2018. Accessed August 14th, 2019. https://www.hcup-us.ahrq.gov/db/state/costtocharge.jsp

[39] Silva J, Tenreyro S. The log of gravity. Rev Econ Stat. 2006;88(4):641-658. doi:10.1162/rest.88.4.641

[40] US Department of Labor, Bureau of Labor Statistics. Consumer price index. Accessed May 8, 2015. https://www.bls.gov/cpi/home.htm

[41] Clapp MA, Little SE, Zheng J, Robinson JN. A multi-state analysis of postpartum readmissions in the United States. Am J Obstet Gynecol. 2016;215(1):113.e1-113.e10. doi:10.1016/j.ajog.2016.01.174 27829570

[42] Fridman M, Korst LM, Chow J, Lawton E, Mitchell C, Gregory KD. Trends in maternal morbidity before and during pregnancy in California. Am J Public Health. 2014;104(suppl 1):S49-S57. doi:10.2105/AJPH.2013.301583 24354836 PMC4011109

[43] Janakiraman V, Lazar J, Joynt KE, Jha AK. Hospital volume, provider volume, and complications after childbirth in US hospitals. Obstet Gynecol. 2011;118(3):521-527. doi:10.1097/AOG.0b013e31822a65e4 21826039

[44] Xu X, Gariepy A, Lundsberg LS, . Wide variation found in hospital facility costs for maternity stays involving low-risk childbirth. Health Aff (Millwood). 2015;34(7):1212-1219. doi:10.1377/hlthaff.2014.1088 26153317

[45] Nguyen PL, Gu X, Lipsitz SR, . Cost implications of the rapid adoption of newer technologies for treating prostate cancer. J Clin Oncol. 2011;29(12):1517-1524. doi:10.1200/JCO.2010.31.1217 21402604 PMC3082972

[46] Matlock DD, Groeneveld PW, Sidney S, . Geographic variation in cardiovascular procedure use among Medicare fee-for-service vs Medicare Advantage beneficiaries. JAMA. 2013;310(2):155-162. doi:10.1001/jama.2013.7837 23839749 PMC4021020

[47] Thomas MP, Parzynski CS, Curtis JP, . Percutaneous coronary intervention utilization and appropriateness across the United States. PLoS One. 2015;10(9):e0138251. doi:10.1371/journal.pone.0138251 26379053 PMC4575022

[48] Medicaid and CHIP Payment and Access Commission. Medicaid payment initiatives to improve maternal and birth outcomes. MACPAC Issue Brief. April 2019. Accessed August 9th, 2019. https://www.macpac.gov/wp-content/uploads/2019/04/Medicaid-Payment-Initiatives-to-Improve-Maternal-and-Birth-Outcomes.pdf

[49] TennCare. 2017 episodes of care results. Created 2017. Accessed August 9th, 2019. https://www.tn.gov/content/dam/tn/tenncare/documents2/TennCareEpisodes2017Results.pdf

[50] Kozhimannil KB, Graves AJ, Ecklund AM, Shah N, Aggarwal R, Snowden JM. Cesarean delivery rates and costs of childbirth in a state Medicaid program after implementation of a blended payment policy. Med Care. 2018;56(8):658-664. doi:10.1097/MLR.0000000000000937 29912840

[51] Ayres-De-Campos D, Cruz J, Medeiros-Borges C, Costa-Santos C, Vicente L. Lowered national cesarean section rates after a concerted action. Acta Obstet Gynecol Scand. 2015;94(4):391-398. doi:10.1111/aogs.12582 25783672

[52] Dranove D. Pricing by non-profit institutions. The case of hospital cost-shifting. J Health Econ. 1988;7(1):47-57. doi:10.1016/0167-6296(88)90004-5 10302654

[53] Woodworth L, Romano PS, Holmes JF. Does insurance status influence a patient’s hospital charge? Appl Health Econ Health Policy. 2017;15(3):353-362. doi:10.1007/s40258-017-0308-z 28164250 PMC5429205

[54] Muhlestein D. What types of hospitals have high charge-to-reimbursement ratios? Health Affairs *blog*. Posted July 15, 2013. Accessed February 12, 2021. doi:10.1377/hblog20130715.033072

[55] Bai G, Anderson GFUS. US hospitals are still using chargemaster markups to maximize revenues. Health Aff (Millwood). 2016;35(9):1658-1664. doi:10.1377/hlthaff.2016.0093 27605648

