# Supplementary material for: Evaluation of Hospital Cesarean Delivery–Related Profits and Rates in the United States
Source: JAMA Netw Open. 2021 Mar 19;4(3):e212235. doi: 10.1001/jamanetworkopen.2021.2235 (PMC7980096; doi:10.1001/jamanetworkopen.2021.2235)
Supplement: Supplement. — eTable. Frequencies and Percentages Associated With Maternal Comorbidities, Delivery-Related Procedures, Delivery Complications, and Postpartum Complications [file jamanetwopen-e212235-s001.pdf]

Supplemental Online Content

Sakai-Bizmark R, Ross MG, Estevez D, et al. Evaluation of hospital cesarean delivery–related profits and rates in the United States. *JAMA Netw Open*. 2021;4(3):e212235. doi:10.1001/jamanetworkopen.2021.2235

eTable. Frequencies and Percentages Associated With Maternal Comorbidities, Delivery-Related Procedures, Delivery Complications, and Postpartum Complications

This supplemental material has been provided by the authors to give readers additional information about their work.

© 2021 Sakai-Bizmark R et al. *JAMA Network Open.*

**eTable 1**: **Frequencies and percentages associated with maternal comorbidities, delivery-related procedures, delivery complications, and postpartum complications**						
		**Profit Quartiles**				
	**Total (n = 13,215,853)**	**Quartile 1 (n = 2,356,544)**	**Quartile 2 (n = 3,400,263)**	**Quartile 3 (n= = 4,090,553)**	**Quartile 4 (n = 3,368,493)**	***P***
**Maternal Comorbidities**						
Asthma	480,010 (3.6%)	81,778 (3.5%)	114,998 (3.4%)	160,314 (3.9%)	122,921 (3.6%)	0.04
Diabetes	839,877 (6.4%)	134,588 (5.7%)	209,645 (6.2%)	260,482 (6.4%)	235,162 (7.0%)	<0.001
Drug Dependency/Substance Abuse	203,627 (1.5%)	48,680 (2.1%)	48,905 (1.4%)	61,075 (1.5%)	44,967 (1.3%)	<0.001
Hypertensive Disorder	1,160,759 (8.8%)	192,133 (8.2%)	308,162 (9.1%)	385,959 (9.4%)	274,505 (8.1%)	0.90
Obesity	618,099 (4.7%)	98,605 (4.2%)	137,214 (4.0%)	203,472 (5.0%)	178,808 (5.3%)	<0.001
Psychiatric Disease	484,691 (3.7%)	101,459 (4.3%)	124,925 (3.7%)	154,696 (3.8%)	103,611 (3.1%)	<0.001
Seizure Disorder	50,807 (0.4%)	9,226 (0.4%)	12,901 (0.4%)	16,670 (0.4%)	12,010 (0.4%)	0.11
Smoking	281,948 (2.1%)	41,427 (1.8%)	66,769 (2.0%)	104,638 (2.6%)	69,114 (2.1%)	0.002
Thyroid Disease	373,147 (2.8%)	68,506 (2.9%)	91,846 (2.7%)	120,617 (2.9%)	92,178 (2.7%)	0.53
**Delivery-Related Procedures**						
Forceps Delivery	100,142 (0.8%)	19,569 (0.8%)	36,814 (1.1%)	28,756 (0.7%)	15,003 (0.4%)	<0.001
Vacuum Delivery	709,341 (5.4%)	131,500 (5.6%)	166,865 (4.9%)	220,642 (5.4%)	190,333 (5.7%)	0.03
Operative Delivery (Unspecified)	185 (0.0%)	52 (0.0%)	45 (0.0%)	51 (0.0%)	37 (0.0%)	0.11
Induction of Labor	3,400,245 (25.7%)	613,146 (26.0%)	969,292 (28.5%)	1,055,818 (25.8%)	761,989 (22.6%)	<0.001
Episiotomy	1,131,826 (8.6%)	197,377 (8.4%)	299,224 (8.8%)	352,312 (8.6%)	282,914 (8.4%)	0.77
Hysterectomy	3,953 (0.0%)	525 (0.0%)	999 (0.0%)	1,363 (0.0%)	1,066 (0.0%)	<0.001
Transfusion	104,100 (0.8%)	18,491 (0.8%)	27,239 (0.8%)	31,176 (0.8%)	27,193 (0.8%)	0.84
Dilation and Curettage	41,267 (0.3%)	7,471 (0.3%)	10,297 (0.3%)	12,833 (0.3%)	10,666 (0.3%)	0.72
Laparotomy	2,560 (0.0%)	503 (0.0%)	716 (0.0%)	799 (0.0%)	542 (0.0%)	0.01
**Delivery Complications**						
Hemorrhage	856,185 (6.5%)	166,735 (7.1%)	218,364 (6.4%)	266,828 (6.5%)	204,258 (6.1%)	0.02
Infection	55,956 (0.4%)	8,946 (0.4%)	14,384 (0.4%)	18,051 (0.4%)	14,575 (0.4%)	0.008
Laceration	1,108,964 (8.4%)	193,946 (8.2%)	299,773 (8.8%)	355,364 (8.7%)	259,881 (7.7%)	0.004
Operative Injury	8,048 (0.1%)	1,179 (0.1%)	2,014 (0.1%)	2,646 (0.1%)	2,209 (0.1%)	0.002
Thrombotic Event	97,382 (0.7%)	18,018 (0.8%)	25,510 (0.8%)	29,269 (0.7%)	24,585 (0.7%)	0.36
Uterine Rupture	0 (0.0%)	0 (0.0%)	0 (0.0%)	0 (0.0%)	0 (0.0%)	-

© 2021 Sakai-Bizmark R et al. *JAMA Network Open.*

Other	73,362 (0.6%)	12,216 (0.5%)	19,762 (0.6%)	22,945 (0.6%)	18,440 (0.5%)	0.73
**Postpartum Complications**						
Acute Cardiovascular Disease (including cardiomyopathy, heart failure, myocardial infarction)	1,995 (0.0%)	289 (0.0%)	510 (0.0%)	683 (0.0%)	512 (0.0%)	0.09
Acute Cerebrovascular Disease (including stroke and intracranial hemorrhage)	1,646 (0.0%)	290 (0.0%)	341 (0.0%)	536 (0.0%)	479 (0.0%)	0.08
Anesthesia Complications	39,280 (0.3%)	7,909 (0.3%)	10,215 (0.3%)	11,801 (0.3%)	9,355 (0.3%)	<0.001
Appendicitis	582 (0.0%)	133 (0.0%)	138 (0.0%)	174 (0.0%)	137 (0.0%)	0.23
Bacteremia, Sepsis	2,885 (0.0%)	389 (0.0%)	637 (0.0%)	984 (0.0%)	875 (0.0%)	<0.001
Gallbladder Disease	58,910 (0.4%)	8,966 (0.4%)	13,597 (0.4%)	18,467 (0.5%)	17,880 (0.5%)	<0.001
Non-specific Postpartum Diagnosis	305,099 (2.3%)	51,405 (2.2%)	76,247 (2.2%)	96,769 (2.4%)	80,677 (2.4%)	0.002
Mastitis, Breast Abscess	1,503 (0.0%)	227 (0.0%)	449 (0.0%)	485 (0.0%)	342 (0.0%)	0.74
Pancreatitis	2,177 (0.0%)	341 (0.0%)	544 (0.0%)	752 (0.0%)	540 (0.0%)	0.31
Upper Respiratory Infection	10,509 (0.1%)	1,862 (0.1%)	2,679 (0.1%)	3,513 (0.1%)	2,456 (0.1%)	0.40
Urinary Tract Infection (including pyelonephritis)	120,947 (0.9%)	20,934 (0.9%)	31,235 (0.9%)	38,075 (0.9%)	30,702 (0.9%)	0.66
Uterine Infection	55,995 (0.4%)	7,715 (0.3%)	13,679 (0.4%)	18,841 (0.5%)	15,760 (0.5%)	<0.001
Wound Infection and/or Breakdown	42,406 (0.3%)	7,921 (0.3%)	10,156 (0.3%)	13,857 (0.3%)	10,472 (0.3%)	0.78
Death	375 (0.0%)	69 (0.0%)	100 (0.0%)	118 (0.0%)	86 (0.0%)	0.61

© 2021 Sakai-Bizmark R et al. *JAMA Network Open.*
